# The Effect of Aggregated Alpha Synuclein on Synaptic and Axonal Proteins in Parkinson’s Disease—A Systematic Review

**DOI:** 10.3390/biom12091199

**Published:** 2022-08-29

**Authors:** Jennifer Murphy, Declan P. McKernan

**Affiliations:** Pharmacology & Therapeutics, School of Medicine, National University of Ireland, H91 TK33 Galway, Ireland

**Keywords:** Parkinson’s disease, α-synuclein, synaptic proteins, axonal motor proteins, neurodegeneration

## Abstract

α-synuclein is a core component of Lewy bodies, one of the pathological hallmarks of Parkinson’s disease. Aggregated α-synuclein can impair both synaptic functioning and axonal transport. However, understanding the pathological role that α-synuclein plays at a cellular level is complicated as existing findings are multifaceted and dependent on the mutation, the species, and the quantity of the protein that is involved. This systematic review aims to stratify the research findings to develop a more comprehensive understanding of the role of aggregated α-synuclein on synaptic and axonal proteins in Parkinson’s disease models. A literature search of the PubMed, Scopus, and Web of Science databases was conducted and a total of 39 studies were included for analysis. The review provides evidence for the dysregulation or redistribution of synaptic and axonal proteins due to α-synuclein toxicity. However, due to the high quantity of variables that were used in the research investigations, it was challenging to ascertain exactly what effect α-synuclein has on the expression of the proteins. A more standardized experimental approach regarding the variables that are employed in future studies is crucial so that existing literature can be consolidated. New research involving aggregated α-synuclein at the synapse and regarding axonal transport could be advantageous in guiding new treatment solutions.

## 1. Introduction

In addition to dementia with Lewy bodies (DLB) and multiple system atrophy (MSA), Parkinson’s disease (PD) is often described as a ‘synucleinopathy’ [1,2]. Synucleins are a family of proteins with three distinct members, including alpha, beta, and gamma synuclein [3]. Alpha synuclein (α-synuclein) is a natively unfolded, 140 amino acid protein which is encoded by the *snca* gene on human chromosome 4 [4,5,6]. The aggregates of α-synuclein form a major component of the proteinaceous inclusions called Lewy bodies, which are commonly found in nigral, limbic, and brainstem nuclei in PD [2,7,8].

Despite continuous investigation, the precise physiological functions of α-synuclein are somewhat elusive [1]. Research has shown that α-synuclein localizes primarily to the presynaptic terminals and adopts a chaperone-like function, mediating exocytosis, synaptic vesicle (SV) formation and functionality, and neurotransmitter release [4,9,10]. α-synuclein also may promote soluble N-ethylmaleimide-sensitive factor attachment protein receptor (SNARE)-complex assembly by binding directly to the SNARE protein vesicle-associated membrane protein 2 (VAMP-2) [11]. The SNARE complex itself is comprised of the proteins VAMP-2, synaptosomal-associated protein 25 (SNAP-25) and syntaxin-1 [12]. The complex enables the SVs to fuse with the plasma membrane at the active zone (AZ) which in turn enables neurotransmitter release and the assembly and disassembly of the complex prior to and after neurotransmitter release generates the energy that is required to fuel this membrane fusion [12,13]. Pathologically, research has illustrated that several point mutations of the *snca* gene including A30P or A35T are linked to dominant familial PD, which are also risk factors for sporadic PD [14,15]. Furthermore, at the synapse, toxic aggregations, and mutations of α-synuclein can have multiple effects including the loss of presynaptic proteins, decreasing neurotransmitter release, and inhibiting SV recycling [16,17,18,19].

Normal brain function depends on the ability of neurons to communicate both efficiently and quickly with each other at the synapse [20]. There is a vast network of proteins at play here to ensure efficient information flow and intracellular connectivity (see Table 1) and each protein requires the regulation of the others in order for this flow to occur [21]. Moreover, the maintenance of cell homeostasis and communication between the cell body and the periphery, as well as neuronal plasticity and survival relies on efficient and reliable axonal transport [22,23]. Similar to the process of synaptic transmission, active transport in the neuron is also dependent on a network of motor proteins (see Table 2). Neurodegenerative disorders such as Alzheimer’s disease have identified axonal transport impairments as a predominant event in neurodegeneration [24]. However, research investigating axonal transport deficits in PD is more limited [25,26]. Interferences in the axonal transport network can represent early pathology in disorders such as PD [27], with α-synuclein appearing to target the functioning of this network even prior to signs of neurodegeneration [28].

Research on α-synuclein includes the use of post-mortem human tissue, genetic studies, animal models, and the use of cell culture systems [15,53,54]. Post-mortem tissue while very valuable, does not allow for intervention. Also, animal models do not always accurately reflect the human condition. A wide variety of animal models have been used from synuclein overexpression, pesticides, inflammatory insults, and more recently small molecule aggregators, each having their own pros and cons [55,56,57]. Therefore, there are many inconsistencies in the evidence regarding the specific effect of α-synuclein on the expression and function of synaptic and axonal proteins [25,29,30,31,35,40,44]. The discrepancies in the research are linked to the inconsistent approaches of the investigations aiming to dissect the role of α-synuclein toxicity at a cellular level [58]. Identifying the specific impact of α-synuclein toxicity on synaptic and axonal proteins could facilitate researchers in predicting the pattern of events that ensue throughout the brain as a result. This systematic review aims to stratify research findings to develop a more comprehensive understanding of the role of aggregated and mutant α-synuclein specifically at both the site of the synapse and axon in PD. This may in turn allow for the identification of more specific disease biomarkers and potentially therapies for this condition.

## 2. Methods

### 2.1. Search Strategy

To carry out the systematic review, comprehensive literature searches were conducted of PubMed, Web of Science, and Scopus databases. The primary search strategy used Medical Subject Headings (MeSH) and keywords including: “alpha synuclein” OR “synuclein” AND “aggregation” OR “overexpression”; “synapse” OR “synaptic cleft”; AND “axonal proteins” OR “synaptic proteins”. Secondary searches included the following terms which were used in a variety of combinations: “oligomers”, “fibrils”, “monomers”, “Parkinson’s disease”, “synucleinopathy”, “presynaptic”, “postsynaptic”, “endocytosis”, “exocytosis”, “active zone proteins”, “axonal transport”, “retrograde”, and “anterograde”. Moreover, the following specific synaptic and axonal proteins and axonal associated structures were included as secondary search terms to ensure a comprehensive literature search; “SNARE proteins”, “rab proteins”, “synapsin”, “RIM”, “munc-18”, “rabphilin”, “PSD95”, “synaptophysin”, “complexin”, “piccolo/bassoon”, “amphyphysin”, “drebrin”, “kinesin”, “dynein”, “myosin”, “actin”, “microfilaments”, “tubulin”, “KIF1A/1B/2A/3B”, and “microtubules”. In addition, to ensure that all the relevant studies will be included in this review, citation tracing and bibliographic analysis of references in the relevant studies were used. An additional search was carried out every month until July 2022 to ensure that the latest articles that met the criteria were included.

The initial inclusion criteria were as follows: (a) full-text international peer-reviewed articles; (b) studies that were conducted between the period of 1990 and 2022; (c) data that were collected from both human, rodent, or post-mortem samples; (d) published in English; (e) α-synuclein aggregation, overexpression, or mutant α-syn. The initial exclusion criteria included (a) gamma or beta synuclein; (b) the effect of α-synuclein on any structure or molecule other than synaptic or axonal proteins; (c) systematic reviews, meta-analyses, book extracts, and conference proceedings. Following the first literature search for studies relating to axonal proteins and α-synuclein aggregation, an additional inclusion criterion of associated axonal structures (e.g., microtubules, actin filaments, microfilaments) was incorporated to the search.

### 2.2. Study Screening and Selection

**Figure 1:**
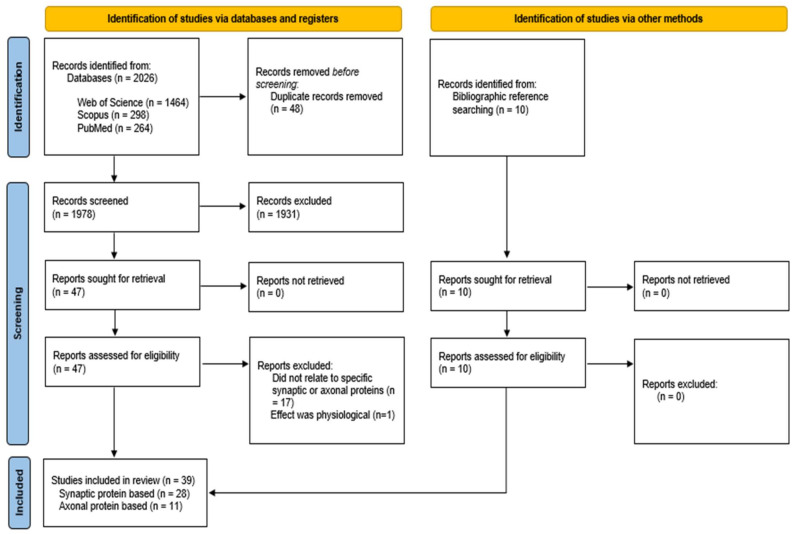
PRISMA flow diagram of the systematic review carried out on the literature.

The abstracts of the results were exported into Abstrackr, an online abstract screening application (Wallace et al., 2012). The imported results were initially screened by title and abstract according to ‘labels’ or key words which included ‘alpha synuclein’, ‘protein’, ‘synaptic’, and ‘axonal’, enabling a more efficient screening process. Duplicate studies were removed. All of the studies that met the inclusion criteria were assessed further with the steps of the selection process that were highlighted in the Preferred Reporting Items for Systematic Reviews and Meta-Analyses (PRISMA) flow diagram (Figure 1) [65]. A total of 2026 abstracts were identified from the databases with 1464 that were obtained from the Web of Science, 298 from Scopus, and 264 from PubMed. There were ten additional abstracts that were identified via bibliographic reference searching. Removing duplicates excluded 48 abstracts and initial screening using the inclusion criteria excluded 1978 abstracts. Of the remaining 57 abstracts, 39 fulfilled the inclusion criteria and provided data regarding α-synuclein toxicity and synaptic and axonal proteins. Of those excluded, 17 did not refer to specific synaptic or axonal proteins and one referred to a physiological α-synuclein effect (Figure 1).

### 2.3. Data Extraction

The data were extracted to a Microsoft Excel spreadsheet and the results were organized according to certain variables including author’s name, year of publication, type of α-synuclein which was further categorized into mutant (e.g., A53T α-syn), species (e.g., oligomeric α-syn), and human (human wild-type α-syn). Additional variables included the sample and species, concentration of α-syn, assay that was used, technique that was used for analysis, quantitative findings, and qualitative main findings. This systematic review was reported according to the PRISMA guidelines and, therefore, followed the PRISMA checklist and flow diagram as mentioned (Figure 1) [65]. Statistical analyses were performed of the general data using Microsoft Excel functions. Comparative statistics were calculated regarding the species of alpha synuclein that was used, the sample from which the data were obtained, and the technique of analysis that was used. There were three key data elements that were selected for specific analysis—sample type, form of α-synuclein that was used, and the effect on any identified synaptic and axonal proteins. The effect that α-synuclein had on synaptic and axonal proteins was analyzed and a table of all the findings was created (see Table 1 and Table 2, respectively). However, a comparison of all the included investigations was not possible due to the high volume of variables in the studies. Therefore, each investigation was analyzed separately and all the investigations that used either the same sample type or form of α-synuclein were isolated and the findings were compared to identify any consistent findings or any trends in the findings.

## 3. Results

### 3.1. Study Characteristics

A total of 39 studies were included in this review, from which 63 individual investigations were isolated for analysis. There were 48 studies that investigated synaptic proteins and 16 studies that investigated axonal proteins. Several techniques of analysis were used in the investigations. For the purpose of this review, the investigations were categorized by the following methods—immunohistochemistry (IHC) (*n* = 20), Western blotting (WB) (*n* = 35), ELISA (*n* = 6), quantitative PCR (QPCR) (*n* = 2), electron microscopy (EM) (*n* = 1), and video-enhanced differential interference contrast microscopy (*n* = 1). In total, 34 individual synaptic proteins and 21 axonal proteins and structures were identified in the investigations.

### 3.2. Sample Sources Used in Investigations

**Figure 2:**
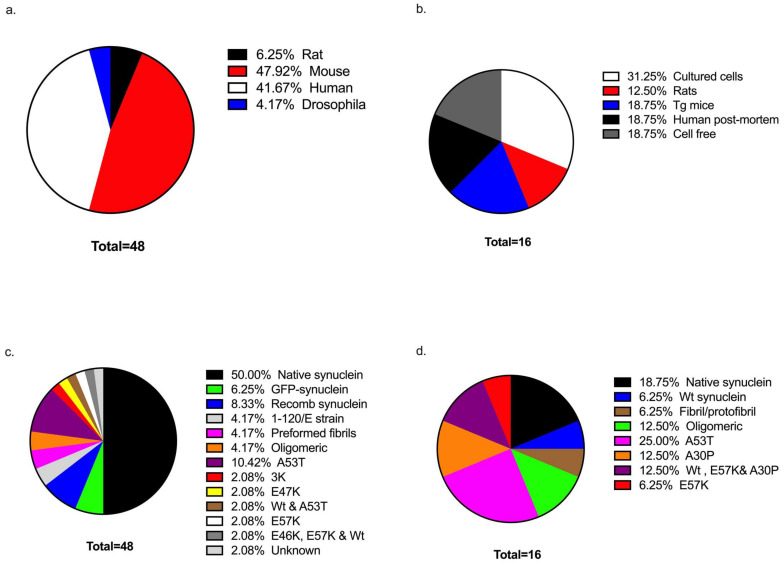
Shown in this figure is a breakdown of the studies that were analyzed in this systematic review to study synaptic proteins (**a**,**c**) and axonal proteins (**b**,**d**). For each protein category (synaptic or axonal), the source of sample for studies (**a**,**b**) and the type of α synuclein that was used in the study (**c**,**d**) are shown.

There were four main sources of sample that were used in the investigations relating to synaptic proteins. Mouse models were employed most frequently (*n* = 23), and human post-mortem tissue was also used (*n* = 20), while the remaining investigations used rats (*n* = 3) and Drosophila (*n* = 2). Of the mouse studies, transgenic mice were used in 65% of the investigations (*n* = 15). The other investigations used mouse hippocampal neurons (*n* = 4), cultured cortical neurons (*n* = 2), and primary embryonic mouse ventral mesencephalic cells (*n* = 2). With regards to the human studies, post-mortem tissue was used in 65% of these investigations (*n* = 13) (Figure 2). Brain tissue was obtained from patients with PD (*n* = 5), dementia with Lewy bodies (DLB) (*n* = 3), PD with dementia (*n* = 1), both PDD and DLB cases (*n* = 3), and both PDD and DLB cases (*n* = 1). The majority of the investigations obtained tissue from the prefrontal cortex (*n* = 7), in addition to the substantia nigra (SN), caudate, and putamen (Table 1). Other human cells of interest that were used in the research involved the human neuroblastoma SH-SY5Y cell line (*n* = 3), induced pluripotent stem cells (iPSC) (*n* = 2), Lund human mesencephalic (LUHMES) cells (*n* = 1), and neural-derived extra-vesicles (NDEs) that were extracted from the peripheral blood of PD patients (*n* = 1). Finally, three investigations employed rat models which included Sprague Dawley rats (*n* = 1), a BAC transgenic rat model (*n* = 1), and rat primary cultured neurons (*n* = 1). Drosophila flies were also used in two investigations (Figure 2).

For studies investigating axonal proteins, human cells were used in 50% of the investigations (Figure 2). This included post-mortem tissue from patients with PD (*n* = 1), PD and DLB cases (*n* = 1), and PD and incidental Lewy Body disease (iLBD) (*n* = 1), as well as LUHMES cells (*n* = 2), human iPSCs (*n* = 1), and human neuroblastoma cells (*n* = 1) (see Table 2). Additionally, mouse models were used in four of the investigations and consisted of the transgenic mouse models (*n* = 3) and mouse embryonic stem (MES) cells (*n* = 1). Moreover, two investigations employed a model of Sprague-Dawley rats (*n* = 2), and three employed a cell-free system (*n* = 3) (Figure 2).

### 3.3. Forms of α-Synuclein

The 48 investigations relating to synaptic proteins in this systematic review employed different forms of α-synuclein. The type of α-synuclein studied included native α-synuclein, aggregate forms of α-synuclein (oligomers, fibrils, etc.), mutant α-synuclein or a combination of mutant, and species-specific forms (Figure 2). Native α-synuclein was studied most frequently in this selection of investigations (*n* = 24). In addition, green fluorescent protein (GFP)-tagged human α-synuclein (*n* = 3) and recombinant human α-synuclein (*n* = 4) were also used. There were two investigations that specifically used strains 1-120 and 1-120E of human α-synuclein (*n* = 2). Regarding the aggregate forms of α-synuclein, recombinant α-synuclein preformed fibrils (PFFs) (*n* = 2) and oligomeric α-synuclein (*n* = 2) were used. Mutant A53T α-synuclein was used most frequently (*n* = 5) where there was a specific point mutation in the protein. 3K α-synuclein (*n* = 1), E47K (*n* = 1), and both wild-type (WT) and A53T α-synuclein (*n* = 1) were also used. There were wo investigations that specifically mentioned the use of E57K oligomers of α-synuclein (*n* = 1) and oligomers of mutants E46K, E57K, and WT α-synuclein (*n* = 1), respectively. One investigation did not specify the form of α-synuclein that was used (*n* = 1).

Several forms of α-synuclein were studied in the 16 studies investigating axonal proteins in this systematic review (Figure 2). Various mutations of α-synuclein were used in the research for example, including α-synuclein mutants A30P (*n* = 2) and A53T (*n* = 4), as well as WT α-synuclein seeds (*n* = 1). Other forms of α-synuclein that were used in studies included oligomeric α-synuclein (*n* = 1) and both oligomeric and monomeric α-synuclein (*n* = 1). Oikawa and colleagues focused research on the use of fibrils and protofibrils of C-terminally truncated α-synuclein [61]. Additionally, Prots and colleagues employed monomers and oligomers of the E57K, A30P, and α-synuclein (30–110) mutants, as well as WT α-synuclein and E57K α-synuclein oligomers [59].

### 3.4. Alpha Synuclein Effects on Synaptic Proteins

Due to the inconsistency in the sample, the type of α-synuclein and technique of analysis that was used across the studies, it was not feasible to compare the findings in a statistically significant manner. Table 1 and Table 2 provide an outline of the results that were found for each protein that was identified. Therefore, this review will discuss trends in the findings and discuss any comparable findings on the basis of sample type or form of α-synuclein that was used.

The proteins VAMP-2, SNAP-25, and synataxin-1 form the aforementioned SNARE complex which is a key component of the workings of the synapse [66]. SNAP-25 was identified in 18 investigations (Table 1), with half of the studies (*n* = 9) indicating that the protein expression levels remained unchanged and six reporting reduced protein levels (*n* = 6). Additionally, an increase in the protein levels and a redistribution of the protein to the striatum of the human brain, as well as in the striatum of α-synuclein (1–120) transgenic mice was identified [17,30]. Synaptophysin is a key protein that is involved in synaptic vesicle recycling and synapse formation [67]. The levels of synaptophysin expression were reported in several studies (*n* = 18) (Table 1). The majority found reduced levels of the protein (*n* = 12), with no change identified in four (*n* = 4) and increased levels found in two investigations (*n* = 2), respectively.

Lim and colleagues reported reduced synaptophysin levels when analyzing the overexpression of the A53T α-synuclein mutation in a sample of postnatal day 14 and 21 forebrain-specific conditional transgenic mice. The levels of SNAP-25 and syntaxin were not significantly affected [32]. They also found similar significant reductions in synaptophysin expression levels when analyzing the expression of A53T mutant α-synuclein in 8 month old transgenic mice models of PDD and DLB using the CaMKII promoter. Similarly, syntaxin and SNAP-25 protein levels remained unchanged [35]. Wihan and colleagues used a sample of 52 week old A53T-overexpressing transgenic mice under the PFGFb promoter. While no change in the levels of the SNAP-25 protein were detected, in contrast to previous investigations, the synaptophysin levels also remained unchanged [36].

In 2010, a study that focused on mouse hippocampal neurons found via ELISA that the overexpression of α-synuclein led to a reduced amount of the synaptophysin protein. The researchers also illustrated that excess α-synuclein led to a reduction in the synaptophysin levels in cultured cortical mouse neurons that were incubated with α-synuclein, with the addition of 10 nM of α-synuclein reduced the protein level by 80% for example [50]. Another study using IHC likewise identified a reduction of 45% in synaptophysin levels in mouse hippocampal neurons when compared to the control littermates [30]. However, treating mouse hippocampal neurons with α-synuclein preformed fibrils led to contrasting results with no change in synaptophysin levels and a significant reduction in the SNAP-25 protein levels [31]. The latter result also contrasts with that of Scott and Roy, which was the only study to identify increased SNAP-25 protein levels, specifically by 12% in comparison to the control littermates.

With regards to the VAMP2 expression, the majority of the investigations reported reduced protein levels (*n* = 10) (Table 1). One report found increased levels, while three other studies indicated that the expression levels remained unchanged [45]. One group found a redistribution of the protein to the striatum of transgenic mice (Table 1) [17]. A study in 2018 analyzed human neuroblastoma SH-S5Y5 cells and found that both DA-induced oligomeric α-synuclein aggregates and α-synuclein PPFs lead to sequestered levels of VAMP-2 [34]. In line with this, another group detected decreased protein levels of VAMP2, syntaxin-1, and synaptophysin when exposed to overexpressed human WT α-synuclein in transgenic mice around 12 months old, as well as both the E46K and 3K α-synuclein mutations [43]. By contrast however, another group reported no changes in the levels of these proteins, despite also using human WT α-syn and a similar analysis technique [18]. However, the mouse sample that was used was significantly younger at 24 and 36 days old.

Several studies examined changes in syntaxin-1 protein levels (*n* = 8). However, the findings were inconsistent with reports of no change (*n* = 3) and reductions (*n* = 3) in the protein levels, as well as the redistribution of the protein to the striatum of transgenic mice (*n* = 2) when exposed to α-synuclein overexpression and mutations. Hence, there were too many variables to make any definitive conclusions.

Synapsins are key proteins that are involved in synaptogenesis and so a number of studies have investigated the effects of synuclein on its expression [68]. The majority of studies detected reduced or undetectable synapsin expression levels (*n* = 5) (Table 1). In a study using Drosophila for example, the group demonstrated reduced synapsin expression levels using IHC in the synaptic boutons [33]. While measuring the progression of the disease, downregulated synapsin levels were also found using blots on day 3 and on day 20 of the trial. Spinelli and colleagues also found decreased synapsin staining in synaptic terminals of transgenic mice with overexpressed GFP-tagged human α-synuclein [47]. Similarly, another study also compared vacant synapses and non-vacant synapses in the hippocampal neurons of transgenic mice that were overexpressing GFP-tagged human α-synuclein. Vacant synapses did not contain any detectable levels of synapsin, as well as the proteins VAMP2, piccolo, and amphiphysin. The transgenic boutons containing these presynaptic proteins (non-vacant synapses) also contained diminished amounts of the four proteins, with losses of 51%, 30%, 15%, and 17%, respectively, in comparison to WT boutons [19]. Further, another study by the same authors found similarly reduced amphiphysin levels of 30% in mouse hippocampus neurons [30].

By contrast, synapsin III levels were generally found to have increased (*n* = 4) (Table 1). Human post-mortem tissue of PD patients revealed a marked accumulation of synapsin III in the caudate and putamen regions when compared with healthy controls. Additionally, the researchers noted that the protein was redistributed in both transgenic mice and in the primary DA mesencephalic neurons of mice [49]. Similarly, but using quantitative PCR, human brain tissue of PD patients in Braak stages 1–2 revealed increased synapsin III levels in the SN of 45% [44]. Additionally, no alterations in the distribution or expression level of SV2 or SM proteins were detected in transgenic mice [18]. The levels of synaptotagmin-1, a key protein that is involved in fusion and endocytosis [69], also remained unchanged when exposed DA-induced oligomeric α-synuclein aggregates in human neuroblastoma SH-SY5Y cells [34].

Regarding post-synaptic proteins, only two were identified in the included investigations—PSD-95 and neurogranin (see Table 1). PSD-95 regulates synaptic maturation by its interaction with receptors [70] while neurogranin potentiates synaptic transmission [71]. The findings were generally consistent regarding the effect that α-synuclein aggregation had on PSD-95, with levels reduced in the post-mortem tissue of PD (by 48%) [46], and DLB cases (by 28% and 17%, respectively) [52]. In a sample of postnatal day 14 and 21 forebrain-specific conditional transgenic mice overexpressing the A53T mutation of α-syn, the PSD-95 levels were also reduced [32]. By contrast however, another group detected no change in the level of the PSD-95 protein, despite similarly analyzing transgenic mice that were expressing the A53T mutation under the PDGFb promoter [36]. The animals were significantly older in age at 52 weeks old. With regards to neurogranin, the levels of this protein were analyzed in only one human post-mortem sample of patients with Alzheimer’s disease and DLB. However, the researchers used two techniques including WB and ELISA and found a 21–38% and 30–51% decrease in the neurogranin levels, respectively [38].

RIM proteins have a key role in the tethering of synaptic vesicles and their preparation for release [13]. Reduced levels of the RIM3 protein were detected in a BAC transgenic rat sample, as well as of RIMS3 in a transgenic mouse sample specifically of 55% [29,39]. Another similar protein, piccolo, which is involved in guiding of the SVs, has been studied [13]. Reduced piccolo levels were also detected in the hippocampal neurons of transgenic mice overexpressing human WT α-synuclein:GFP. Specifically, a 15% reduction was identified in non-vacant mouse synapses, while the levels in vacant mouse synapses were undetectable [19]. An interacting partner of RIM, Rab3 is also involved in the tethering of SVs [13]. Human post-mortem tissue that was obtained from PDD and DLB patients revealed reduced Rab3A expression levels with a loss of 24–34% identified via WB and a loss of 27–43% identified via the ELISA technique. The research also detected reduced levels in 3k mutant mice [43]. However by contrast, another group found a 1.3-fold increase in the Rab3a protein levels (30%) in transgenic mice that were expressing the A53T α-synuclein mutation [36].

### 3.5. Effects of α-Synuclein on Axonal Proteins

Similar to the findings regarding synaptic proteins and α-synuclein, it was not possible to compare the findings in a statistically significant manner due to the inconsistency in the sample, type of α-synuclein, and technique of analysis that was used across the studies. Therefore, this review will discuss trends in the findings and identify any like-for-like comparisons that can be made. The findings relating to all the axonal proteins and structures that were identified are presented in Table 2.

Conventional kinesin is a major microtubule-based motor protein which is responsible for anterograde axonal transport [25]. Members of the kinesin family of proteins were consistently reduced when they were exposed to the overexpression of and mutations of α-synuclein (Table 2). Chu and colleagues for example found that the immunoreactivity levels of the protein were downregulated even in early-stage PD. Similarly, in a sample of brain tissue from PD and incidental LBD patients, a group found that kinesin light chain protein levels were downregulated in early stages of the disease (Braak stage 1–2) compared with the controls [44]. Regarding the vast family of kinesin proteins, KIF1A, KIF1B, KIF2A, and KIF3A levels were reduced 8 weeks after an injection with adeno-associated virus (AAV2)-A53T α-synuclein in the striatum of Sprague-Dawley rats. The researchers found contrasting results regarding the KIF1A protein, in that the protein levels were increased in the substantia nigra and a reduction in the striatum of the Sprague-Dawley rat model [16]. Another group detected a similar increase in the KIF1A levels in transgenic mice in which the A53T mutant α-synuclein was expressed under the PDGFb promoter [36]. Finally, in 2013, a study found that there was a reduction of acetylated tubulin in cells that were overexpressing WT α-synuclein seeds and E57K α-synuclein oligomers in LUHMES cells via the lentiviral construct. Within the neurites in these cells, there were also decreased amounts of the protein KIF5. The reduction in KIF5, however, was more than in cells that were expressing E57K α-synuclein oligomers compared to the WT seeds [59].

Cytoplasmic dynein is an important retrograde axonal transport motor protein [8]. Findings relating to the effect that is exerted by α-synuclein on levels of the protein are inconsistent (Table 2). For example, a study found severely reduced levels of dynein, specifically dynein light chain Tctex Type 3 (DYNLT3) which is a subunit of this large protein complex in human post-mortem PD tissue in the later stages of the disease [25]. The changes were also inconsistent with regards to animal models. Wihan and colleagues detected unchanged levels of dynein when A53T mutant α-synuclein was expressed in transgenic mice under the PDGFb promoter [36]. In a similar sample of transgenic mice in which the same A53T mutant α-synuclein was overexpressed, the level of the protein was decreased in the striatum, yet significantly increased in the substantia nigra pars compacta [60].

Further inconsistent results were obtained in the investigations that employed the Sprague-Dawley rat models (Table 2). With an injection of AAV2-A53T α-synuclein in the SN of Sprague-Daley rats, the dynein levels as well as the levels of dynamitin and dynactin 1 were increased [16]. Another group also explored the effect of the overexpression via injection of rAAV-h-A30P α-synuclein in the SN of Sprague-Daley rats and contrastingly found a reduction in both the dynein and kinesin levels by IHC [25].

With regards to tubulin, no changes were detected in slow axonal transport including total soluble axonal and cytoskeletal proteins such as tubulin, actin, and the neurofilament triplets (NF-L, NF-M, and NF-H) in preclinical models or in pre-symptomatic PD patients [62]. Another study found that tubulin was redistributed when it was exposed to overexpressed α-synuclein human neuroblastoma SH-SY5Y cells. The researchers also detected no change in the actin microfilament levels, as well as filamentous microtubules when they were exposed to overexpressed α-synuclein human neuroblastoma SH-SY5Y cells [63].

## 4. Discussion

This systematic review offers support for the dysregulation or redistribution of key synaptic proteins as a result of α-synuclein toxicity. However, due to the high quantity of variables that were used across the studies that were analyzed, it was challenging to statistically quantify what effect α-synuclein had on the expression of synaptic proteins. Certain trends were identified. Regarding the SNARE complex machinery, for example, the majority of the findings found a reduction in the VAMP2 protein levels, while studies found reductions or no changes in the SNAP-25 protein levels (Table 1). Specifically, Choi and colleagues illustrated that α-synuclein oligomers sequestered VAMP2, thus blocking SNARE-mediated vesicle docking and potentially disrupting the exocytosis of neurotransmitters [34]. Moreover, the levels of the post-synaptic proteins PSD95 and neurogranin were downregulated in both rodent and human models [38,39,46]. Hence, the findings are indicative that α-synuclein exerts its influence at both pre- and post-synaptic sites. Additionally, the review highlights a potential disturbance in AZ regulation, as associated protein levels appear to be dysregulated due to the presence of toxic α-synuclein. Reduced piccolo protein levels were detected in transgenic mice that were overexpressing α-synuclein [19], inferring a disruption in the guidance of SVs to the AZ [21]. Further, reductions of 24–34% of the Rab3A protein were identified [43]. Rab3A contributes to tethering and docking neurotransmitter vesicles [72].

Furthermore, toxic species and aggregation of α-synuclein lead to changes in axonal proteins expression, thus inferring the occurrence of dysfunctional axonal transport in PD. The levels of the kinesin family proteins for example were consistently reduced [25,44]. Changes relating to the dynein protein, however, were more inconsistent. Reductions in the protein levels were found in the striatum of A53T transgenic mice [60]. Yet, increased protein levels were identified in the substantia nigra pars compacta of these mice [60] and of Sprague-Daley rats [16].

In order to stratify existing research, variables including sample and form of α-synuclein were accounted for in each study, as both play a crucial role in determining how particular research findings are understood. Mouse models were used in the majority of the investigations. Mouse models have a high conservation of basal ganglia circuit with humans for example [73]. However, they cannot fulfil every key neurological and pathological feature of PD [53] and do not provide enough resolution to precisely visualize axonal transport in live neurons or measure the kinetics of axonal transport [8]. Additionally, there may be concerns regarding the age of the animal. The mouse generally has a short-life span of 2 years which can make modelling a neurodegenerative disease challenging [53]. Means of overexpression are generally employed in an attempt to shorten the time of disease manifestation, but this must be interpreted with caution [53]. Correspondingly, the age of the animal that was used was found to be an additional variable factor across the included studies. The synaptophysin levels, for example, remained consistent regardless of the age of the mice that were used, with samples from prenatal days 14 and 21 [32], 8 months [35], and 52 weeks reporting reduced levels of the protein [36]. By contrast, mice measuring 12 months old illustrated both increased and decreased Rab3a expression levels [36,43]. Hence, such findings tend to be validated using human post-mortem tissue, which is often classed as the gold standard against which the ability of animal models to reflect accurate disease markers is assessed [53]. Indeed, human post-mortem tissue was the second most commonly used sample type, which logically appears an effective choice. However, post-mortem investigations must be interpreted with caution as disease heterogeneity and the interval between death and post-mortem examination could influence the results [25]. Within this review, for example, several studies used tissue from PD and DLB cases, which made it difficult to correlate findings to specific disease subgroups.

Moreover, experimental evidence has previously and specifically pointed towards the more detrimental effect of oligomeric α-synuclein [74,75]. Indeed, oligomeric α-synuclein was most commonly used, with only two studies referring to the use of α-synuclein preformed fibrils. Oligomeric forms of α-synuclein were found to exert a more detrimental effect on proteins compared to monomeric and fibrillar forms [74]. α-synuclein E57K and A30P oligomers, for example, decreased the gliding velocity of microtubules, whereas monomeric forms exerted no influence [59]. The findings support existing research that illustrates the link between oligomeric E57K α-synuclein and a more severe dopaminergic loss in the SN, compared to fibrillar forms [76]. Evidently, different forms of α-synuclein exert varying degrees of toxicity. The discrepancy in research methodology makes it difficult to ascertain precisely what effect α-synuclein toxicity has on synaptic or axonal proteins. Ultimately, future studies need to have a stratified and standardized approach to consolidate existing findings and to develop a more comprehensive understanding of the pathogenic role of α-synuclein at a cellular level.

Currently, the treatment for PD is limited, with the dopaminergic medication Levodopa the gold standard in the treatment of motor-related symptoms [77]. However, the treatment only offers motor-related symptom relief which declines in efficacy as the disease progresses [78]. While deep brain stimulation methods have shown promise in relieving symptoms and improving quality of life [77], this treatment similarly provides symptomatic relief without addressing neuron degeneration for example, nor the debilitating non-motor expression of the disease such as cognitive decline. Thus, research needs to focus on a new path and on treatments that target and potentially halt such progressive degenerative process [79].

There are over 70 Rab proteins which act as “master regulators” of the membrane trafficking network and ensure that cargo is delivered to its correct destinations via numerous effector proteins [22,80]. While the research is still limited, there has been evidence that the overexpression of certain Rab proteins can curb pathogenic α-synuclein toxicity [81]. Experimental evidence has indicated that proteins Rab3A [72], Rab8b, Rab11a, and Rab13 [82] promote the clearance of α-synuclein aggregates, thereby preventing α-synuclein-induced toxicity. Additionally, α-synuclein monomers and oligomers cooperatively inhibit neuronal SNARE-mediated vesicle fusion and at sub-micromolar concentrations, α-synuclein monomers increased the fusion inhibition by α-synuclein oligomers [83]. The researchers developed a model to block oligomer binding and thus reduce toxicity during vesicle fusion. These research findings illustrate the value of research into the intricate processes underlying the alteration of synaptic and axonal proteins by pathogenic α-synuclein and highlight the potential these revelations have in formulating new treatments that could target the neurological dysfunctions that are faced by those that are suffering with PD, rather than just offering them motor-based symptomatic-relief.

Evidently, α-synuclein toxicity impacts synaptic processes via the alteration of crucial synaptic proteins. Disturbances in synaptic homeostasis leads to a cascade of synaptic misfiring, degeneration, the spreading of α-synuclein pathology, and a lack of DA provision which is detrimental to the survival of the neuron and underlie the motor symptoms that are experienced by PD patients. Further, impaired retrograde axonal transport of autophagy-related organelles could create a pathogenic feedback loop of defective axonal transport [8]. Increases in the α-synuclein levels correlate with decreases in autophagy-related proteins at the lysosome and in the proteosome [25] which promote cell survival by eliminating protein aggregates and defective organelles including excess α-synuclein [84,85]. Dysfunctions in this process could contribute to the aggregation of toxic α-synuclein, a failure to detect the misfolding, fibrillization and aggregation of pathogenic α-synuclein and ultimately synaptic loss and cell death [8,25]. Therefore, with an abundance of synaptic, axonal and scaffolding proteins, and adaptors that regulate the intracellular communication and transport of specific cargo along the axons [8], exploring the early functional changes to susceptible proteins, neurons, and circuits that occur in the earliest stages of PD could prove revolutionary in identifying these new paths in PD treatment.

With regards to limitations, firstly there were too many variables in the studies that were included in this review which made it challenging to ascertain what effect α-synuclein had quantitatively on the expression of synaptic and axonal proteins. As addressed briefly, there is a need for a standardized approach to investigations relating to this research question, so that previous findings can be affirmed or denied and new insights into the mechanisms behind α-synuclein toxicity at the synapse and during axonal transport can be uncovered. Additionally, while an exhaustive literature search was conducted, it is possible that some studies were not included in the review that would have offered further insight into the effect that is exerted by α-synuclein aggregation on synaptic and axonal proteins. Future reviews would benefit from employing as many combinations of key words and phrases as possible during the literature search to be assured that all potential articles have been identified.

This review supports the hypothesis that the aggregation and mutations of α-synuclein dysregulates or redistributes synaptic and axonal transport proteins. However, due to the inconsistency in the investigations, it was not possible to ascertain the precise effect that α-synuclein toxicity imposes on the proteins that were identified. The review acknowledges the need for a more standardized approach regarding the variables that are employed in future studies such as the form of α-synuclein that is used or the sample type that is chosen for example, so that existing literature can be consolidated, and new insights gained. Ultimately, there is a need for a new treatment approach that focuses on targeting the underlying mechanisms of PD such as neuronal degeneration, rather than those that just provide symptomatic relief. New research involving aggregated α-synuclein at the synapse and in relation to axonal transport could be advantageous in offering these treatment solutions.

## Figures and Tables

**Table 1:** The effect of α-synuclein on presynaptic and postsynaptic proteins. Shown in this table are the individual synaptic proteins that are affected by aggregated α-synuclein. In each case, the effect, quantity of change (if available), sample source, model, technique, and reference are given.

Protein	Effect	Quantity of Change	Sample	Model	Technique	Reference
**SNAP-25**	No change		Mice	mThy-1 α-syn E57K Tg mouse and mThy-1 α-syn WT mouse models	WB	[29]
	Increased ↑	12%	Mice	Overexpression of α-syn in cultured mouse hippocampal neurons	IHC	[30]
	Reduced ↓		Rats	Primary hippocampal neuronal culture treated with α-syn-hWT PFFs	WB	[31]
	No change		Mice	Truncated human α-syn, strains (1-120) and (1-120E) expressed in Tg mice	WB	[17]
	Redistributed		Human	Post-mortem tissue from the straitum of PD patients	IHC	[17]
	Redistributed		Mice	Truncated human α-syn, strains (1-120) and (1-120E) expressed in Tg mice	IHC	[17]
	No change		Mice	Forebrain-specific conditional Tg mice overexpressing human WT or A53T	WB	[32]
	No change		Drosophila flies	Drosophila model expressing human α-syn	IHC	[33]
	Reduced ↓		Human	DA-induced oligomeric α-syn aggregates in human neuroblastoma SH-SY5Y	WB	[34]
	Reduced ↓		Rats	Primary cultured neurons treated with α-syn aggregates	WB	[34]
	No change		Mice	A53T mutant α-syn were expressed in Tg mice models of PDD and DLB	IHC	[35]
	No change		Mice	Tg mice expressing the A53T mutant α-syn under the PDGFb promoter	WB	[36]
	No change		Human	NDEs from peripheral blood of PD patients	WB	[37]
	Reduced ↓	24–42%	Human	Post-mortem tissue from the PFC, BA21, BA24, and BA40 of PDD and DLB patients	WB	[38]
	Reduced ↓	15–48%	Human	Post-mortem tissue from the PFC, BA21, BA24, and BA40 of PDD and DLB patients	ELISA	[38]
	No change		Rats	BAC Tg rat model overexpressing the full-length human SNCA	WB	[39]
	Reduced ↓		Human	α-syn oligomer-forming mutants E46K and E57K and WT α-syn expressed in human iPSC-derived neurons	WB	[40]
	No change		Mice	Overexpression of human WT α-syn in Tg mice	WB	[18]
**SNAP-29**	Reduced ↓		Human	Post-mortem tissue from the SNpc of PD patients	IHC	[41]
	Reduced ↓		Human	α-syn transduced LUHMES cells via adenoviruses serotype 5 (AV5)	WB	[41]
**SNAP-23**	Reduced ↓		Human	α-syn transduced LUHMES cells via adenoviruses serotype 5 (AV5)	WB	[41]
**SNAP-47**	Reduced ↓	24%	Human	Post-mortem tissue from the PFC of PD patients	ELISA	[42]
**VAMP2**	Reduced ↓	31%	Mice	Overexpression of α-syn in cultured mouse hippocampal neurons	IHC	[30]
	No change		Mice	Overexpression of human WT α-syn in Tg mice	WB	[18]
	Reduced ↓		Mice	Primary hippocampal neuronal culture treated with α-syn PPFs	WB	[31]
	Redistributed		Mice	Truncated human α-syn, strains (1-120) and (1-120E) expressed in Tg mice	IHC	[17]
	Reduced ↓		Mice	A53T mutant α-syn were expressed in Tg mice models of PDD and DLB using the CaMKII promoter	IHC	[35]
	Undetectable		Mice	Hippocampal neurons of Tg mice overexpressing human α-syn:GFP	IHC	[19]
	Reduced ↓	30%	Mice	Hippocampal neurons of Tg mice overexpressing human α-syn:GFP	IHC	[19]
	Reduced ↓	30%	Human	NDEs from peripheral blood of PD patients	WB	[37]
	No change		Drosophila flies	Drosophila model expressing human α-syn	IHC	[33]
	Reduced ↓		Human	DA-induced oligomeric α-syn aggregates in human neuroblastoma SH-SY5Y	WB	[34]
	Reduced ↓		Rats	Primary cultured neurons treated with α-syn aggregates	WB	[34]
	Reduced ↓		Mice	Tg mice expressing 3K α-syn mutation	WB	[43]
	Reduced ↓		Human	Post-mortem tissue from the SN of PD patients (Braak stages 5–6)	QPCR	[44]
	Reduced ↓		Human	Post-mortem tissue from the PFC of DLB patients	WB	[45]
**Syntaxin**	Reduced ↓		Rats	Nigral injection of AAv2-A53T α-syn	WB	[16]
	Reduced ↓		Mice	Forebrain-specific conditional Tg mice overexpressing human WT or A53T mutant α-syn	WB	[32]
	Redistributed		Mice	A53T mutant α-syn were expressed in Tg mice models of PDD and DLB using the CaMKII promoter	IHC	[35]
	No change		Human	α-syn was overexpressed in LUHMES cells via adenoviruses serotype 5 (AV5)	WB	[41]
	Reduced ↓		Drosophila flies	Drosophila model expressing human α-syn	WB	[33]
	Reduced ↓	57%	Human	iPSCs from patients with the A53T α-syn mutation	WB	[46]
**Synataxin 1**	No change		Mice	Truncated human α-syn, strains (1-120) and (1-120E) expressed in Tg mice	WB	[17]
	Reduced ↓		Human	Post-mortem tissue from the PFC of DLB patients	WB	[45]
	Redistributed		Human	Post-mortem tissue from the striatum of PD patients	IHC	[17]
	Redistributed		Mice	Truncated human α-syn, strains (1-120) and (1-120E) expressed in Tg mice	IHC	[17]
	Reduced		Mice	Tg mice expressing 3K α-syn mutation	WB	[43]
	Reduced		Human	Post-mortem tissue from the SN of PD patients (Braak stages 5-6)	QPCR	[44]
	No change		Mice	Primary hippocampal neuronal culture treated with α-syn PFFs	WB	[31]
	No change		Mice	Overexpression of human WT α-syn in Tg mice	WB	[18]
**Syntaxin 1a**	No change		Human	DA-induced oligomeric α-syn aggregates in human neuroblastoma SH-SY5Y	WB	[34]
	No change		Rats	Primary cultured neurons treated with α-syn aggregates	WB	[34]
	Reduced ↓	20%	Human	NDEs from peripheral blood of PD patients	WB	[37]
**Synaptotagmin**	No change		Mice	Overexpression of human WT α-syn in Tg mice	WB	[18]
	Reduced ↓		Mice	A53T mutant α-syn were expressed in Tg mice models of PDD and DLB using the CaMKII promoter	IHC	[35]
	No change		Drosophila flies	Drosophila model expressing human α-syn	IHC	[33]
**Synaptotagmin 1**	No change		Human	DA-induced oligomeric α-syn aggregates in human neuroblastoma SH-SY5Y	WB	[34]
**Synaptotagmin 2**	Reduced ↓	19%	Human	Post-mortem tissue from the PFC of PD patients	WB	[42]
	Reduced ↓	24%	Human	Post-mortem tissue from the PFC of DLB patients	WB	[42]
	Increased ↑		Human	Post-mortem tissue from the SN of PD patients (Braak stages 1–2)	QPCR	[44]
**SM proteins**	No change		Mice	Overexpression of human WT α-syn in Tg mice	WB	[18]
**Complexin**	Reduced ↓		Mice	Tg mice expressing 3K α-syn mutation	WB	[43]
**Complexin 2**	Reduced ↓		Human	Post-mortem tissue from the SN of PD patients (Braak stages 5–6)	QPCR	[44]
	Reduced ↓		Mice	Overexpression of human WT α-syn in Tg mice	WB	[18]
**Synapsin**	Undetectable		Human	Post-mortem tissue from the frontal cortical sections of the brain from DLB patients	IHC	[19]
	Reduced ↓		Drosophila flies	Drosophila model expressing human α-syn	IHC	[33]
	Reduced ↓		Drosophila flies	Drosophila model expressing human α-syn	WB	[33]
	Reduced ↓		Mice	GFP-tagged human α-syn was overexpressed in Tg mice	IHC	[47]
	Reduced ↓		Mice	Forebrain-specific conditional Tg mice overexpressing human WT or A53T mutant α-syn	WB	[32]
	Reduced rate ↓		Mice	Human α-syn transduced via pET17b vector into hippocampal neurons to assess synapsin condensate formation	WB	[48]
**Synapsins**	Reduced ↓		Mice	Overexpression of human WT α-syn in Tg mice	WB	[18]
**Synapsin I**	Reduced ↓	30–50%	Mice	mThy-1 α-syn E57K Tg mouse and mThy-1 α-syn WT mouse model	WB	[29]
	No change		Human	Human neuroblastoma SH-SY5Y cells expressing human α-syn	WB	[49]
	No change		Mice	Primary embryonic mouse ventral mesencephalic cells expressing aggregated α-syn via glucose deprivation	WB	[49]
	Reduced ↓		Mice	A53T mutant α-syn expressed in Tg mice models of PDD and DLB using the CaMKII promoter	IHC	[35]
	Undetectable		Mice	Hippocampal neurons of Tg mice overexpressing human α-syn:GFP	IHC	[19]
	Reduced ↓	51%	Mice	Hippocampal neurons of Tg mice overexpressing human WT α-syn:GFP	IHC	[19]
	Reduced ↓		Human	α-syn oligomer-forming mutants E46K and E57K and WT α-syn expressed in human iPSC-derived neurons	WB	[40]
	Reduced ↓	43%	Mice	Overexpression of α-syn in cultured mouse hippocampal neurons	IHC	[30]
	No change		Mice	Tg mice expressing the A53T mutant α-syn under the PDGFb promoter	WB	[36]
	Reduced ↓		Human	Post-mortem tissue from the SN of PD patients (Braak stages 5–6)	QPCR	[44]
	Reduced ↓		Rats	BAC Tg rat model overexpressing the full-length human SNCA	WB	[39]
**Synapsin II**	Reduced ↓		Mice	Primary hippocampal neuronal culture treated with α-syn PFFs	WB	[31]
**Synapsin III**	Increased ↑		Human	Post-mortem tissue from the caudate and putamen of PD patients	IHC	[49]
	Increased ↑		Mice	Primary embryonic mouse ventral mesencephalic cells expressing aggregated α-syn via glucose deprivation	WB	[49]
	Increased ↑		Human	Human neuroblastoma SH-SY5Y cells expressing human α-syn	WB	[49]
	Redistributed		Mice	Expression of a C-terminally truncated form of human α-syn (1–120) in Tg mice	IHC	[49]
	Redistributed		Mice	Primary embryonic mouse ventral mesencephalic cells expressing aggregated α-syn via glucose deprivation	IHC	[49]
	Increased ↑		Human	Post-mortem tissue from the SN of PD patients (Braak stages 1–2)	QPCR	[44]
**Synaptophysin**	Reduced ↓	45%	Mice	Overexpression of α-syn in cultured mouse hippocampal neurons	IHC	[30]
	No change		mice	Overexpression of human WT α-syn in Tg mice	WB	[18]
	Reduced ↓		Mice	A53T mutant α-syn were expressed in Tg mice models of PDD and DLB using the CaMKII promoter	IHC	[35]
	Reduced ↓		Mice	Tg mice expressing 3K α-syn mutation	WB	[43]
	Reduced ↓	50%	Mice	Cultured cortical neurons incubated with α-syn (500 nM)	ELISA	[50]
	Reduced ↓	80%	Mice	Cultured cortical neurons incubated with α-syn (10 uM)	ELISA	[50]
	Reduced ↓		Mice	Hippocampal neurons with added α-syn	ELISA	[50]
	Reduced ↓		Mice	Forebrain-specific conditional Tg mice overexpressing human WT or A53T mutant α-syn	WB	[32]
	Reduced ↓		Human	α-syn oligomer-forming mutants E46K and E57K and WT α-syn expressed in human iPSC-derived neurons	WB	[40]
	No change		Mice	Tg mice expressing the A53T mutant α-syn under the PDGFb promoter	WB	[36]
	Increased ↑		Human	Post-mortem tissue from the SN of PD patients (Braak stages 5–6)	QPCR	[44]
	Increased ↑		Human	Post-mortem tissue from the SN of PD patients (Braak stages 1–2)	QPCR	[44]
	Reduced ↓		Mice	Overexpression of human α-syn under the mThy1 promoter	IHC	[51]
	No change		Rats	BAC Tg rat model overexpressing the full-length human SNCA	WB	[39]
	Reduced ↓	52%	Human	iPSCs from patients with the A53T α-syn mutation	WB	[46]
	Reduced ↓	20%	Mice	mThy-1 α-syn E57K Tg mouse model	WB	[29]
	Reduced ↓		Human	Post-mortem tissue from the PFC of PDD patients	WB	[45]
	No change		Mice	Primary hippocampal neuronal culture treated with α-syn PFFS	WB	[31]
**SV2C**	Reduced ↓	24%	Human	Post-mortem tissue from the PFC of DLB patients	ELISA	[42]
**SV2**	No change		Mice	Overexpression of human WT α-syn in Tg mice	WB	[18]
**Amphiphysin**	Reduced ↓	30%	Mice	Overexpression of α-syn in cultured mouse hippocampal neurons	IHC	[30]
	Reduced ↓	17%	Mice	Hippocampal neurons of Tg mice overexpressing human WT α-syn:GFP	IHC	[19]
	Undetectable		Mice	Hippocampal neurons of Tg mice overexpressing human WT α-syn:GFP	IHC	[19]
	Reduced ↓		Mice	Tg mice expressing 3K α-syn mutation	WB	[43]
**GAP43**	Reduced ↓	20%	Human	Post-mortem tissue from the PFC of PDD patients	ELISA	[42]
**Rabphilin 3A**	Reduced ↓		Rats	Nigral injection of AAv2-A53T α-syn	WB	[16]
PSD-95 *	Reduced ↓		Mice	Forebrain-specific conditional Tg mice overexpressing the A53T mutant α-syn	WB	[32]
	Reduced ↓		Mice	Tg mice expressing 3K α-syn mutation	WB	[43]
	No change		Mice	Tg mice expressing the A53T mutant α-syn under the PDGFb promoter	WB	[36]
	Reduced ↓		Mice	Overexpression of human α-syn under the mThy1 promoter	IHC	[51]
	Increased ↑		Rats	BAC Tg rat model overexpressing the full-length human SNCA	WB	[39]
	Reduced ↓	48%	Human	iPSCs from patients with the A53T α-syn mutation	WB	[46]
	Reduced ↓	28%	Human	Post-mortem tissue from the PFC, BA24, and BA40 of PDD patients	WB	[52]
	Reduced ↓	17%	Human	Post-mortem tissue from the PFC, BA24, and BA40 of DLB patients	WB	[52]
Drebrin *	Undetectable		Human	iPSCs from patients with the A53T α-syn mutation	WB	[46]
	Reduced ↓		Mice	Forebrain-specific conditional Tg mice overexpressing the A53T mutant α-syn	WB	[32]
Neurogranin *	Reduced ↓	21–38%	Human	Post-mortem tissue from the PFC, BA21, BA24, and BA40 of PDD and DLB patients	ELISA	[38]
	Reduced ↓	30–51%	Human	Post-mortem tissue from the PFC, BA21, BA24, and BA40 of PDD and DLB patients	WB	[38]

Abbreviations: WB: Western blotting; IHC: immunohistochemistry; QPCR: quantitative polymerase chain reaction; Tg: transgenic; PFFs: preformed fibrils; SN: substantia nigra; SNpc: substantia nigra pars compacta; BA21; brain area—temporal lobe neocortex; BA24: brain area—anterior cingulate cortex; BA40: brain area—inferior parietal lobe neocortex; PFC: prefrontal cortex. * Indicates postsynaptic protein.

**Table 2:** Effect of alpha synuclein on axonal proteins and associated structures. Shown in this table are the individual axonal proteins that are affected by aggregated α-synuclein. In each case, the effect, sample source, model, technique, and reference are given.

Protein	Effect	Sample	Model	Technique	Authors
**Kinesin**	Reduced	Human	Post-mortem tissue from the SN of PD patients	IHC	[25]
	Reduced	Rat	Overexpression via injection of rAAV-h-A30P α-syn in SN of rats	IHC	[25]
**Kinesin 1**	Reduced	Human	E46K and E57K oligomer forming mutant α-syn expressed in human iPSC-derived neurons	WB	[40]
**Kinesin light chain**	Reduced	Human	Post-mortem tissue from the SN of PD and iLBD patients (Braak stage 1–2)	QPCR	[44]
**Kinesin Family 20A**	Reduced	Human	Post-mortem tissue from the SN of PD and iLBD patients (Braak stage 1–2)	QPCR	[44]
**KIF1A**	Reduced	Rat	Injection of AAv2-A53T α-syn in striatum of rats (8 weeks)	WB	[16]
	Increased	Rat	Injection of AAv2-A53T α-syn in SN of rats	WB	[16]
	Increased	Mice	A53T mutant α-syn expressed in Tg under the PDGFb promoter	WB	[36]
**KIF1B**	Reduced	Rat	Injection of AAv2-A53T α-syn in striatum of rats (8 weeks)	WB	[16]
**KIF2A**	Reduced	Rat	Injection of AAv2-A53T α-syn in striatum of rats (8 weeks)	WB	[16]
	Increased	Rat	Injection of AAv2-A53T α-syn in SN of rats	WB	[16]
**KIF3A**	Increased	Rat	Injection of AAv2-A53T α-syn in striatum of rats (4 weeks)	WB	[16]
	Reduced	Rat	Injection of AAv2-A53T α-syn in striatum of rats (8 weeks)	WB	[16]
**KIF5**	Reduced	Human	α-syn E57K mutant oligomers overexpressed in LUHMES cells via lentiviral construct	IHC	[59]
	Reduced	Human	WT α-syn seeds overexpressed in LUHMES cells via lentiviral construct	IHC	[59]
	No change	Rat	Injection of AAv2-A53T α-syn in striatum of rats (8 weeks)	WB	[16]
**KIF17**	Reduced	Rat	Injection of AAv2-A53T α-syn in striatum of rats (4 weeks)	WB	[16]
**Dynactin1**	Increased	Rat	Injection of AAv2-A53T α-syn in striatum of rats (8 weeks)	WB	[16]
**Dynamitin**	No change	Rat	Injection of AAv2-A53T α-syn in striatum of rats (4 weeks)	WB	[16]
	Increased	Rat	Injection of AAv2-A53T α-syn in striatum of rats (8 weeks)	WB	[16]
**Dynein**	Reduced	Rat	Injection of AAv2-A53T α-syn in striatum of rats (4 weeks)	WB	[16]
	Increased	Rat	Injection of AAv2-A53T α-syn in striatum of rats (8 weeks)	WB	[16]
	Reduced	Rat	Overexpression via injection of rAAV-h-A30P α-syn in SN of rats	IHC	[25]
	Unchanged	Mice	A53T mutant α-syn expressed in Tg mice under the PDGFb promoter	WB	[36]
	Decreased	Mice	Overexpression of A53T α-syn in striatum of Tg mice	WB	[60]
	Increased	Mice	Overexpression of A53T α-syn in SNpc of Tg mice	WB	[60]
**DYNLT3**	Reduced	Human	Post-mortem tissue from the SN of PD patients	IHC	[25]
**Myosin Va**	Increased	Rat	Injection of AAv2-A53T α-syn in striatum of rats (4 weeks)	WB	[16]
	Reduced	Rat	Injection of AAv2-A53T α-syn in striatum of rats (8 weeks)	WB	[16]
**MT assembly (tau promoted)**	Reduced	Cell free system	WT α-syn expressed in a cell free system (*Escherichia coli*).	EM	[59]
		Cell free system	C-terminally truncated α-syn fibrils and protofibrils subcloned into pRK172 and expressed in a cell-free system (*Escherichia coli* BL21 (DE3))	WB	[61]
**MT gliding**	Reduced	Cell free system	E57K and A30P oligomers of α-syn expressed in cell-free system (*Escherichia coli*).	M	[59]
**Tubulin**	Reduced	Human	α-synuclein E57K mutant oligomers overexpressed in LUHMES cells via lentiviral construct	IHC	[59]
	Reduced	Human	Post-mortem tissue from the SN of PD and iLBD patients (Braak stage 5–6)	QPCR	[44]
	Reduced	Human	WT α-syn seeds overexpressed in LUHMES cells via lentiviral construct	IHC	[59]
	No change	Mice	Tg mice expressing mutant A53T α-syn	WB	[62]
	Redistributed	Human	Overexpression of α-syn via recombinant adenoviral vector in SH-SY5Y human neuroblastoma cells	IHC	[63]
**Tubulin (non-polymerised)**	Increased	Mice	MES cell treated with oligomeric α-syn	WB	[64]
Neurofilament triplets *	No change	Mice	Tg mice expressing mutant A53T α-syn	WB	[62]
**Actin**	No change	Mice	Tg mice expressing mutant A53T α-syn	WB	[62]
	Increased	Rat	Injection of AAv2-A53T α-syn in striatum of rats (4 weeks)	WB	[16]
	Increased	Rat	Injection of Aav2-A53T α-syn in striatum of rats (8 weeks)	WB	[16]
**Actin microfilaments**	No change	Human	Overexpression of α-syn via recombinant adenoviral vector in SH-SY5Y human neuroblastoma cells	IHC	[63]
**MTs (filamentous)**	Reduced	Human	Overexpression of α-syn via recombinant adenoviral vector in SH-SY5Y human neuroblastoma cells	IHC	[63]

Abbreviations: I: immunostaining; B: blotting; Tg: transgenic; SN; Substantia nigra; MT: microtubules; EM: electron microscopy; QPCR; quantitative PCR. * Neurofilament triplets include neurofilament-L (NFL), neurofilament-M (NFM) and neurofilament-H (NFH).

## Data Availability

Not applicable.
